# Influence of Biopsy Technique on Molecular Genetic Tumor Characterization in Non-Small Cell Lung Cancer—The Prospective, Randomized, Single-Blinded, Multicenter PROFILER Study Protocol

**DOI:** 10.3390/diagnostics10070459

**Published:** 2020-07-06

**Authors:** Maik Haentschel, Michael Boeckeler, Irina Bonzheim, Florian Schimmele, Werner Spengler, Franz Stanzel, Christoph Petermann, Kaid Darwiche, Lars Hagmeyer, Reinhard Buettner, Markus Tiemann, Hans-Ulrich Schildhaus, Rainer Muche, Hans Boesmueller, Felix Everinghoff, Robert Mueller, Bijoy Atique, Richard A. Lewis, Lars Zender, Falko Fend, Juergen Hetzel

**Affiliations:** 1 Department of Medical Oncology and Pneumology, Eberhard Karls University, 72076 Tübingen, Germany; Michael.boeckeler@med.uni-tuebingen.de (M.B.); werner.spengler@med.uni-tuebingen.de (W.S.); felix.everinghoff@gmx.de (F.E.); Robert.mueller@med.uni-tuebingen.de (R.M.); Naushad-Bijoy.atique@med.uni-tuebingen.de (B.A.); Lars.Zender@med.uni-tuebingen.de (L.Z.); Juergen.hetzel@med.uni-tuebingen.de (J.H.); 2 Institute of Pathology and Neuropathology, Reference Center for Haematopathology University Hospital, Tuebingen Eberhard-Karls-University, 72076 Tübingen, Germany; Irina.Bonzheim@med.uni-tuebingen.de (I.B.); hans.boesmueller@med.uni-tuebingen.de (H.B.); Falko.Fend@med.uni-tuebingen.de (F.F.); 3 Department of Internal Medicine, Gastroenterology and Tumor Medicine, Paracelsus Hospital, 73760 Ostfildern-Ruit, Germany; f.schimmele@medius-kliniken.de; 4 Center for Pneumology, 58675 Hemer, Germany; franz.stanzel@lkhemer.de; 5 Department for Pulmonary Diseases, Asklepios-Klinik Harburg, 21075 Hamburg, Germany; c.petermann@asklepios.com; 6 Department of Interventional Pneumology, Ruhrlandklinik, University Hospital Essen, University of Duisburg-Essen, 45239 Essen, Germany; kaid.darwiche@rlk.uk-essen.de; 7 Clinic for Pneumology and Allergology, Center of Sleep Medicine and Respiratory Care, Hospital Bethanien Solingen, 42699 Solingen, Germany; lars.hagmeyer@klinik-bethanien.de; 8 Institute of Pathology, University Hospital of Cologne, 50937 Cologne, Germany; reinhard.buettner@uk-koeln.de; 9 Institute for Hematopathology Hamburg, 22547 Hamburg, Germany; mtiemann@hp-hamburg.de; 10 Department of Pathology, University Medicine Essen—Ruhrlandklinik, University Duisburg-Essen, 45147 Essen, Germany; hans-ulrich.schildhaus@uk-essen.de; 11 Institute of Epidemiology and Medical Biometry, Ulm University, 89075 Ulm, Germany; Rainer.muche@uni-ulm.de; 12 NPARU, University of Worcester, Worcester WR2 6AJ, UK; lewisr@doctors.org.uk; 13 Division of Pulmonology, Cantonal Hospital Winterthur, 8400 Winterthur, Switzerland

**Keywords:** NSCLC, molecular genetic characterization, bronchoscopy, cryobiopsy, forceps biopsy, next generation sequencing

## Abstract

The detection of molecular alterations is crucial for the individualized treatment of advanced non-small cell lung cancer (NSCLC). Missing targetable alterations may have a major impact on patient’s progression free and overall survival. Although laboratory testing for molecular alterations has continued to improve; little is known about how biopsy technique affects the detection rate of different mutations. In the retrospective study detection rate of epidermal growth factor (EGFR) mutations in tissue extracted by bronchoscopic cryobiopsy (CB was significantly higher compared to other standard biopsy techniques. This prospective, randomized, multicenter, single blinded study evaluates the accuracy of molecular genetic characterization of NSCLC for different cell sampling techniques. Key inclusion criteria are suspected lung cancer or the suspected relapse of known NSCLC that is bronchoscopically visible. Patients will be randomized, either to have a CB or a bronchoscopic forceps biopsy (FB). If indicated, a transbronchial needle aspiration (TBNA) of suspect lymph nodes will be performed. Blood liquid biopsy will be taken before tissue biopsy. The primary endpoint is the detection rate of molecular genetic alterations in NSCLC, using CB and FB. Secondary endpoints are differences in the combined detection of molecular genetic alterations between FB and CB, TBNA and liquid biopsy. This trial plans to recruit 540 patients, with 178 evaluable patients per study cohort. A histopathological and molecular genetic evaluation will be performed by the affiliated pathology departments of the national network for genomic medicine in lung cancer (nNGM), Germany. We will compare the diagnostic value of solid tumor tissue, lymph node cells and liquid biopsy for the molecular genetic characterization of NSCLC. This reflects a real world clinical setting, with potential direct impact on both treatment and survival.

## 1. Background

Lung cancer is one of the most common cancers, with 230,000 new diagnoses per annum in the United States and 410,000 in Europe, with a prevalence of approximately 1.8 million patients worldwide [1,2,3,4]. Non-small cell lung cancer (NSCLC) accounts for 75–85% of all lung cancers [5]. Two thirds of these patients are diagnosed in non-curable stage III or IV, and are usually treated systemically [6,7,8]. During the last decade, the systemic treatment has developed from a monomorphic platinum-based chemotherapy to a broad spectrum of individually tailored therapeutic approaches, based on the molecular tumor characterization of each patient [9]. NSCLC characterization is aligned to the existing specific therapeutics, but there are now rapidly developing new treatment options [10]. Patients’ individualized treatment becomes more effective, not only because of the broader therapeutic spectrum, but also with the increase of progression free and overall survival (in certain situations) [11,12,13,14,15,16,17,18]. In addition, side effect profiles are better for most targeted drugs compared to the earlier chemotherapy [12,13,14,19,20,21]. However, detection of a distinct mutation, aberration or translocation of the tumor cells and the evidence of a specific immunoreactivity, as determined by the expression of programmed death-receptor 1 (PD-1) and its ligand (PD-L1) on tumor cell surface (PD-L1) or patients’ T-cells (PD-1), are crucial to predict therapeutic effectiveness. So, the detection of a targetable structure predicts therapeutic response and therefore opens up the therapeutic window that would otherwise stay closed.

As a consequence, precise and correct characterization of the molecular genetic profile in patients with non-curable advanced stage III and IV NSCLC is crucial for ensuring optimal treatment; missing any targetable alteration may result in suboptimal therapy and could even impair patients’ overall outcome. Molecular genetic tumor characterization may also be of importance in earlier tumor stages.

Although tumor analytics are claimed to be refined and sensitized down to a single cell level, thus enabling facilitated tumor detection and characterization in the peripheral blood [22], the clinical relevance of this approach analyzing a huge number of cells is unclear. Furthermore, the detection of a single tumor cell type does not necessarily reflect the tumor, with its inherent heterogeneity, since NSCLC tumor heterogeneity has been known and characterized in various studies for a long time [23,24,25,26,27,28,29,30,31,32,33,34]. However, such considerations have rarely been addressed in therapeutically oriented studies. More stringent tumor tissue requirements as a basis for tumor characterization have, up to now, been only vaguely specified or not specified at all, even in large studies or international guidelines [10,35,36,37]. So, there is a definite need to optimize tissue analytics to provide representative tumor characterization; but there is also currently a lack of a ‘gold standard’ for evaluation of this approach [38]. Although large tumor specimens are generally preferred for mutation assays [36]; the required sample size has not been evaluated in the past.

Cryobiopsy (CB) has already been confirmed to be superior to forceps biopsy (FB) for histopathological evaluation of endobronchial malignancies in a prospective study [39]. A retrospective study [40] has confirmed that there is a significant increase in epidermal growth factor (EGFR) detection rate for CB compared to FB, but CB and FB have not yet been compared in terms of molecular genetic tumor characterization. Therefore, we have designed the PROFILER study to compare both bronchoscopic tissue sampling techniques (CB and FB), transbronchial needle aspiration (TBNA) and liquid biopsy for their diagnostic yield in NSCLC.

## 2. Methods/Design

### 2.1. Trial Design

**Figure 1:**
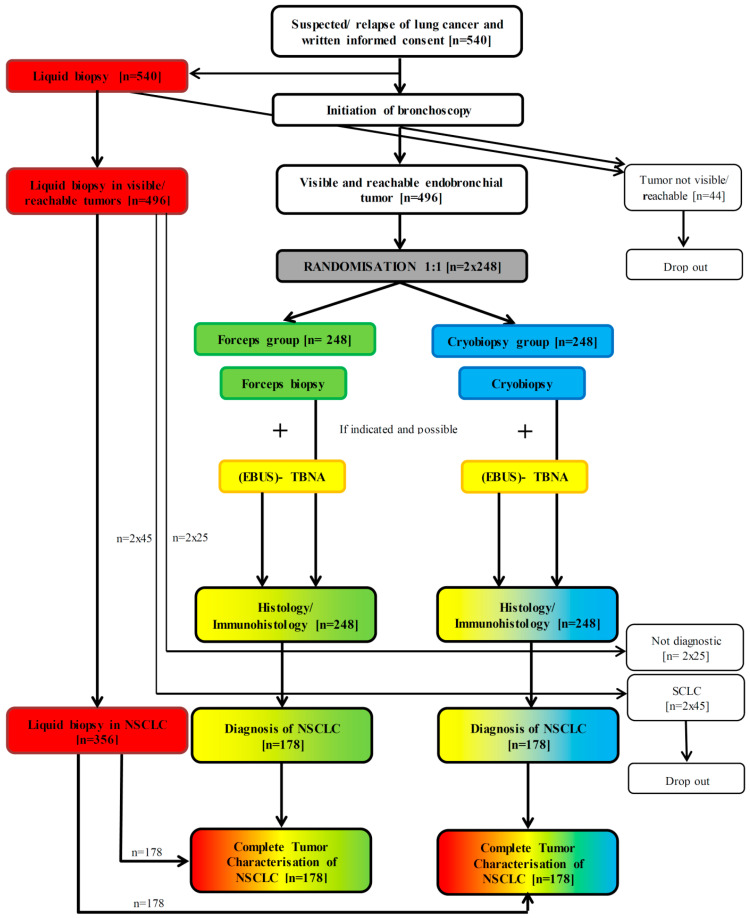
Flow chart of applied techniques and procedures. Assignment of colors: forceps biopsy—green; cryobiopsy—blue; (EBUS-) TBNA—yellow; liquid biopsy—red; (EBUS-) TBNA—endobronchial ultrasound guided transbronchial needle aspiration; NSCLC—non-small cell lung cancer; SCLC—small cell lung cancer.

The PROFILER study is a prospective, multicenter, randomized, single blinded trial in accordance with the German Medical Association Professional Code. It has been designed to evaluate the accuracy of molecular genetic characterization of NSCLC, with the primary aim being to detect the best biopsy technique for tumor cell profiling. Five university hospitals or lung cancer centers across Germany will participate in the study. The enrolment of 356 evaluable patients is planned (Figure 1) with 178 patients having either CB or FB. Based on the study protocol Version 2.9, this study is currently recruiting (the first subject being enrolled on 19 December 2018). The PROFILER study has been registered on www.clinicaltrials.gov; NCT03971175.

### 2.2. Patient Selection

Patients with primary diagnosis of suspected, centrally localized lung cancer, or patients with known NSCLC and suspected relapse after therapy, form the study population. Inclusion and exclusion criteria are shown in Table 1A,B.

### 2.3. Study Objectives

**Figure 2:**
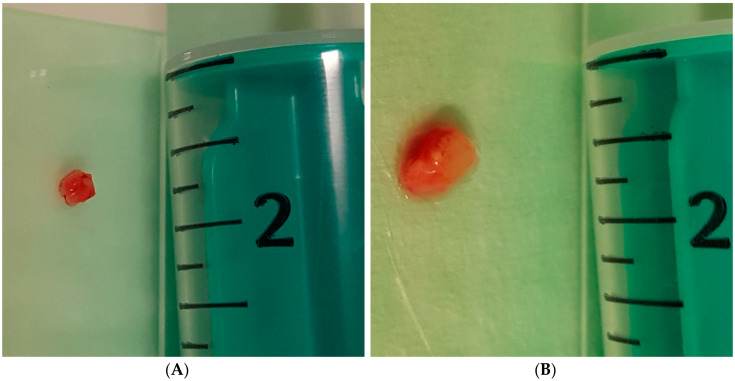
Examples of bronchoscopic forceps biopsy (**A**) and bronchoscopic cryobiopsy (**B**), as shown in two of the study patients.

The primary objective is to assess differences in detection of molecular genetic alterations in NSCLC between FB and CB (Figure 2).

The secondary objectives assess differences in the detection of molecular genetic alterations in NSCLC between liquid biopsy, solid tumor tissue biopsy obtained by bronchoscopic techniques, cytological material obtained by TBNA, and to compare combined methods (tissue biopsy, TBNA and liquid biopsy) with single techniques, to evaluate differences between naïve and processed tumor tissue specimens (e.g., by microdissection), and finally, to assess differences in side effects (e.g., peri-interventional bleeding). 

The exploratory objectives analyze tumor mutational burden with regard to solid tumor tissue by FB or CB and cytologic material by TBNA and liquid biopsy.

### 2.4. Pre-Procedural Training and Standardization

All centers are already expert in the use of both forceps and cryobiopsy. Due to the integration or affiliation to the national network for genomic medicine lung cancer in Germany (nNGM), all participating pathologists will use the same standard for evaluation. 

### 2.5. Procedural Protocol

#### 2.5.1. Patient Enrollment 

The local investigators pre-screen any potential participant with pulmonary lesions which were suspicious of lung cancer, or known NSCLC with suspected relapse or progression, in accordance with the inclusion and exclusion criteria; after giving informed consent, patients are eligible for study entry. Bronchoscopy is undertaken according to local standards, and in accordance with the published guidelines [41,42]. If endobronchial tumor is visible and reachable, inclusion criterion 4 is fulfilled, and the patient can be included in the study. A study flow chart is shown in Figure 1.

#### 2.5.2. Randomization

Patients are randomized to either FB or CB for tissue sampling by a stratified, balanced (1:1) block randomization, stratified by study site. The randomization will be prepared at the Institute of Epidemiology and Medical Biometry, University of Ulm. Sealed envelopes will be prepared for use in every study site, containing the randomized group. The opening of an envelope has to be documented in detail on the case report form (CRF). Liquid biopsy is performed in every patient included in the study, and TBNA is performed if indicated, depending on local investigators’ decision, irrespective of randomization.

#### 2.5.3. Liquid Biopsy Procedure

Prior to any bronchoscopic intervention, blood is drawn for liquid biopsy analysis.

#### 2.5.4. Bronchoscopy

Bronchoscopy is performed using either a flexible or rigid technique, depending on the operator’s choice. In the case of flexible bronchoscopy, placement of an endotracheal tube is recommended in order to provide a secure airway. When rigid bronchoscopy is used, a tissue biopsy is carried out using a flexible bronchoscope inserted through the rigid tube. General anaesthesia will be used for rigid bronchoscopy, and deep sedation and local anaesthesia for intubation with a flexible tube. Patients will be monitored for oxygen saturation, ECG and repeated non-invasive blood pressure, according to the local standards of each center.

#### 2.5.5. Forceps and Cryobiopsy Procedure

Depending on randomization, forceps or cryobiopsy are performed, in accordance with the guidelines and previous reports [39,41,42,43,44]. Forceps of a diameter between 1.8 and 2.6 mm are used for FB; cryoprobes of 1.9 or 2.4 mm (Erbe Elektromedizin GmbH, Tübingen, Germany) are used for CB. Freezing time for CB is dependent on the individual situation, morphology, localization, size and probe positioning, and is determined by the local investigator. A minimum of four biopsies will be taken.

#### 2.5.6. TBNA Procedure

If indicated, TBNA is performed, preferentially under EBUS- guidance, to evaluate hilar and mediastinal lymph node metastasis. In some cases, TBNA may be done without EBUS guidance. EBUS equipment is not predefined by the study protocol; the procedure being performed according to the local standard and guidelines [45,46].

### 2.6. Pathological Analysis

#### 2.6.1. Baseline Pathological Evaluation

The histological, immunohistological and immunohistochemical evaluation of extracted solid tissues by FB or CB, and the cytological, immunocytological and immuncytochemical evaluation of TBNA extracted cytoblock or smear, are performed at the local pathological institute (including ALK, ROS 1 and PD-L1 expression level on tumor cells). Histological and immunohistological tissue evaluation, or cytological and immunocytological evaluation, assigns the tumor tissue to either NSCLC or small cell lung cancer (SCLC). Any cases of SCLC will be excluded from further analyses and classified as a ‘drop out’. For any NSCLC, further molecular genetic evaluation will be undertaken, as described below.

Harmonization of the analytical processes is guaranteed in the study setting by the choice of the individual institutes of pathology, which are partners in the German national network of genomic medicine lung cancer (NGM lung cancer) [47], or which have participated in national collaborative ring trials. Additional collaborative ring trials for the analytical process will be performed for a selection of samples prior to the planned analytical process in this study.

#### 2.6.2. Molecular Genetic Evaluation

Molecular genetic testing will be performed for solid bronchoscopically extracted material, for the cytological specimen extracted by TBNA (if applicable), and for the liquid biopsy samples. All currently targetable and non-targetable genetic aberrations, as suggested by the guidelines [10,35,36,37] (mutations, fusions, copy number variation (CNV)), as well as other important prognostic markers, are analyzed in accordance with the guidelines for standardized evaluation from the nNGM lung cancer consortium [48]. Targeted multigene mutation screening will be performed using different next generation sequencing techniques and platforms, depending on the participating site:

Tübingen: Amplicon library preparation will be done using the nNGM Panel 1.0 and 2.0. Semiconductor sequencing will be performed according to the manufacturers’ manuals using the Ion AmpliSeq Library Kit v2.0, the Ion 510 and Ion 520 and Ion 530 Kit—Chef and the Ion 520 Chip Kit on the Ion GeneStudio S5 (Ion Reporter software) (Thermo Fisher Scientific, Waltham, MA, USA). Fusion and CNV detection will be undertaken with immunohistochemistry following fluorescent in situ hybridization, or with the Archer^®^ FusionPlex Lung panel (Archer Analysis software, ArcherDX, Boulder, CO, USA).

Hamburg: The NEOselect assay (NEO New Oncology, Cologne, Germany) is used to detect single nucleotide mutations, copy number variations and fusions on the Illumina NextSeq system (Illumina, San Diego, CA, USA), according to the manufacturers’ manual. In the case of very limited material, CNVs will be tested using PCR technologies such as cobas^®^; fusion and CNV detection will be undertaken with immunohistochemistry.

Köln: The amplification of DNA will be performed using the customized GeneRead DNAseq custom Panel V2 with primers for the nNGM Panel 1.0 and 2.0 (Qiagen, Hilden, Germany), following the manufacturer’s instructions. For library preparation, the Gene Read DNA Library I Core Kit and the Gene Read DNA I Amp Kit (Qiagen) will be used. After end-repair and adenylation, libraries were ligated to NEXTflex DNA Barcodes (Bio Scientific, Austin, TX, USA) and sequenced on the MiSeq (Illumina, San Diego, CA, USA) with a MiSeq reagent kit V2 (300 cycles) (Illumina), following the manufacturer’s recommendations. Data will be exported as FASTQ files. Alignment, variant calling and annotation will be done using an in-house bioinformatic pipeline. A 5% cutoff for variant calls will be used, and results will only be interpreted if the coverage was >200×. The detection of gene fusions will either be done by a combination of immunohistochemistry and fluorescence in situ hybridization (FISH), or by using the Archer^®^ FusionPlex Lung panel with the Archer Analysis software (ArcherDX, Boulder, CO, USA). CNVs will be analyzed by FISH.

Essen: The QIASeq Targeted DNA Panels (Qiagen, Hilden, Germany) nNGML1 and/or nNGML1.2 [48] will be run on an Illumina MiSeq or Illumina NextSeq platform, and the CLC Genomics Workbench versions 5.0.1, 12.0.3 or 20 will be used for data acquisition. Fusion and CNV detection will be carried out by immunohistochemistry, FISH, or by using Archer^®^ FusionPlex CTL panel (ArcherDX, Boulder, CO, USA).

### 2.7. Safety Data

#### 2.7.1. Bleeding

Bleeding is the most relevant side effect of any bronchoscopic biopsy and is categorized as shown in Table 2.

#### 2.7.2. Other side Effects

In addition to bleeding, hypoxia, cardiac arrhythmia, fever, infection and any other event attributable to the procedure will be evaluated.

### 2.8. Statistical Methodology 

All analyses will be performed by the independent Institute of Epidemiology and Medical Biometry, Ulm University, after database lock and unblinding. A comprehensive statistical analysis plan (SAP) will be prepared prior to a first analysis of the study data.

#### 2.8.1. Sample Size Estimate

Sample size calculation is based on the comparison of the detection rates for both techniques (FB and CB). Based on our earlier retrospective study, the cryobiopsy detection rate of EGFR was increased by approximately 50%, from 14% to 21% [40]. As our primary endpoint combines various aberrations, including PD-L1-expression level, a rate of detectable alterations of around 40% could be expected. With an expected relative increase of almost 50% for aberrations, related to a frequency of 40%, an absolute detection frequency of 55% was considered to be a conservative estimate.

The following assumptions were made for the sample size calculation: two-sided Chi-square test, type one error alpha = 0.05, power = 0.80. With a detection rate of 40% in the control FB group, and a conservative estimation of the detection rate of 55% in the experimental CB-group, and an assumption of equal number of patients in each group, 173 patients are required for each group, i.e., 2 × 173 = 346 patients in the whole trial.

Because of the short duration of observation in each patient, the rate of loss to follow up is very small. To allow a drop-out rate of up to 3% 10 extra patients need to be recruited, resulting in a total sample size of 356 patients. As seen in Figure 1, the sample size for recruitment has to be increased, because NSCLC is likely to form 80% of all tumors; about 45 will be small cell lung cancer (SCLC) and about 10% (25 cases) would not be diagnostic in each cohort. Therefore, 2 × 248 patients will be required for randomisation. Although there is no current data, it can be assumed that in some cases an endobronchial tumor will not be visible. As a rough estimate, we assume that in 8% of the cases (*n* = 44), it will not be possible to take a biopsy from the central airways. This therefore determines that the total number of recruited suspected lung cancer patients is 540.

Sample size calculation was performed by nQuery Advanced 8.1.

#### 2.8.2. Randomization

The equality of study arms will be achieved by a stratified, balanced (1:1) block randomization. Randomization will be stratified according to study site. The randomization will be undertaken independently at the Institute of Epidemiology and Medical Biometry, University of Ulm, using the randomization software ROM. Sealed envelopes are prepared for use in each study site, which contain the randomized group.

#### 2.8.3. Blinding

The study is single blinded, with the pathologists being blinded for the applied biopsy technique.

#### 2.8.4. Primary Endpoint Analysis

The primary endpoint is the detection of at least one molecular genetic alteration in NSCLC. Confirmatory and exploratory data analyses are performed for this dichotomous primary endpoint as follows: 

The difference in both groups based on the primary endpoint are evaluated using a Chi-square test (two sided at a significance level of 5%). All further analyses of the primary endpoint will be performed in an exploratory fashion. All results from these analyses will be regarded as hypothesis-generating, and not as proof of efficacy. Univariate and multiple logistic regression models to adjust for strata (study site) and potential confounding variables will be performed.

#### 2.8.5. Secondary Endpoint Analysis

The secondary, exploratory objectives and safety data are analysed by statistical methods for comparison of two unpaired groups, as appropriate for the characteristic of the variables, e.g., chi-square test for categorical and *t*-test/Mann–Whitney U test for continuous variables. The assessment of the diagnostic value of liquid biopsy and TBNA, as both single techniques and in combination, will be undertaken by logistic regression analysis and ROC analysis. In order to investigate effects of subgroups and putative confounding factors, further analyses are performed, using generalized linear regression models as appropriate.

### 2.9. Trial Oversight and Ethics Approval

All procedures including FB, CB, TBNA and blood extraction for liquid biopsy are standardized routine and approved techniques using CE certified medical devices for diagnosis of lung cancer. The diagnostic workflow and its results are analyzed in this study setting, but they will not be altered at all, so all diagnostic procedures are performed as in routine clinical practice. This study protocol follows the German Medical Association Professional Code with institutional review board (IRB)-approval of the local IRB for each investigational site.

This clinical study is conducted in accordance with all appropriate laws and regulations, including, but not limited to, the Guideline for Good Clinical Practice (GCP), the Code of Federal Regulations (CFR), Directive 2001/20/EC, and the ethical principles of the Declaration of Helsinki and its amendments. Nothing in this document limits the authority of a physician to provide emergency medical care as appropriate.

This study was approved by the leading ethics committee at the University hospital of Tuebingen (644/2018BO1) between 29 August 2018 and 12 December 2018, and approved by local ethics committees at all participating study sites.

## 3. Discussion

As with many other malignancies, NSCLC cannot be considered as a single, monomorphic disease, but rather as a general term for a variety of lung malignancies with different causes, behaviors and prognoses which we are beginning to unravel. Existing and upcoming treatment options for many specified subtypes target identical molecular genetic changes/characteristics as the ‘Achilles’ heel’ of the tumor. Such treatments have dramatically changed management and prognosis of patients over the last decade. However, to enable this increasingly individualized therapy, the exact characterization of each underlying tumor is crucial. Without detection of a specific aberration, e.g. a sensitizing EGFR mutation, the optimal targeted therapy would be ineffective [49,50,51]. On the other hand, missing a molecular genetic aberration, even in a subset of tumor cells, has a relevant and large impact on individual patients’ treatment and prognosis.

Current diagnostic approaches are intended to address this central point by their steadily increasing sensitivity and specificity, ability to analyze the smallest tumor fragments and cytological material or cell free DNA on a single cell level. In addition, there is a desire for easier tumor material extraction, preferentially from the blood. Although this approach contradicts earlier guidelines for NSCLC analysis [35,36], recent updates have focused on analytical techniques, displacing the role of tissue patterns to the background [10,37]. Tumor heterogeneity may be considered by such approaches, but the finding of any or several NSCLC clones does not guarantee that they represent the entire tumor. 

Our previous observations have shown significant differences in EGFR detection between tumor tissue samples extracted by various techniques in a retrospective analysis [40]. This raises the question as to whether the diagnostic yield from different tissue or cytological sampling techniques differs, and if so, to what extent? The diagnostic value for each sampling technique alone has often been addressed [52,53,54,55,56,57,58,59,60,61,62,63,64], but, to our knowledge, differences in NSCLC diagnoses have only been analyzed between different techniques or biopsy sites [65,66], but not in a prospective multicenter study that includes a large study cohort.

This PROFILER study approach uses the basic principles of tumor diagnostics, but takes into account that even the best available diagnostic tool may only be as good as the starting material to be analyzed [35,36]. In general, larger tumor specimens (e.g., resections) are preferred for mutation assays, because of a greater amount of material and a greater capacity to enrich the malignant content by dissection (as discussed in the molecular testing guideline by Lindeman et al.) [36] In the PROFILER study, we compare the complete tumor characterization between FB and CB standard bronchoscopic techniques as the primary endpoint. This concept goes far beyond existing comparisons [67,68,69,70,71,72,73], focusing on the adequacy and representative nature of all cell containing specimens that can be acquired for NSCLC diagnostics, since this has never been addressed in a prospective multicenter trial setting of this size. These results allow determination of which single technique or a combination of techniques best enables NSCLC tumor characterization.

Our study has some potential limitations that have to be addressed. The randomization to FB or CB could lead to an imbalance of the two cohorts, e.g., in terms of patient characteristics, tumor growth or stage, although the sample size should enable a good balance. The histological, cytological and molecular genetic evaluations are not undertaken by a single reference center; they are performed by different local pathologic institutes, using standardized, but sometimes different, analytic sets/kits. However, the multicenter approach for bronchoscopy and the pathological analyses enables the results to be more generalizable and represents a ‘real world’ setting.

Bleeding is the main side effect of bronchoscopic biopsy in visible endobronchial tumors. No difference has been shown in clinically relevant bleeding between FB and CB in prospective trials [39,74].

With this study’s focus on NSCLC diagnostics, we expect to obtain the essential information needed to optimize therapy, and anticipate that the findings will have an impact on both disease progression and survival in many NSCLC patients.

## Figures and Tables

**Table 1:** (**A**) shows Inclusion criteria of the study; (**B**) shows Exclusion criteria of the study.

**(A) Inclusion Criteria**
Informed consent to the study and the study-specific procedures prior to any study intervention
Age ≥ 18 years
Patients with known NSCLC and suspected relapse after therapy
Patients with known NSCLC and suspected relapse after therapy
Bronchoscopically visible tumor
**(B) Exclusion Criteria**
Pre-existing malignancy other than NSCLC
Contraindication for bronchoscopy follow the international guidelines [41,42], daily clinical practice and local regulations, and exclude: - Patients with existing or at risk of pulmonary and cardiovascular decompensation - Patients at increased risk of bleeding with antiplatelet agents except aspirin (e.g., clopidogrel, ticlopidine), anticoagulant therapy (abnormal PTT), thrombocytopenia (<50.000/uL) or coagulopathy (abnormal in-vitro bleeding time) - Intolerance to sedation - Unstable or immobile cervical spine - Limited motion of the temporomandibular joint - Previous enrolment in the present study

**Table 2:** Periinterventional bleeding.

Category	Intervention for Bleeding Control
No	Self-limiting bleeding without need for any intervention for bleeding control
Mild	Self-limiting bleeding, manageable with suction alone and without the need for any specific intervention
Moderate	Non self-limiting bleeding with need for suction plus additional intervention (alone or in combination) including application of ice-cold saline or vasoconstrictors, or transient balloon tamponade, leading to a termination of bleeding
Severe	Non self-limiting bleeding with need for suction, plus any additional intervention (alone or in combination) and need for prolonged observation, stay in the hospital or intensive care therapy
Persistent/Fatal	Any persisting health impairment or death as a direct consequence of bleeding

## Data Availability

The datasets generated and/or analyzed during the current study are not publicly available, in accordance with the ethics committee’s decision, but may be available from the corresponding author on reasonable request and with the ethics committee’s consent.
